# Reduction in Inter-Hemispheric Connectivity in Disorders of Consciousness

**DOI:** 10.1371/journal.pone.0037238

**Published:** 2012-05-22

**Authors:** Smadar Ovadia-Caro, Yuval Nir, Andrea Soddu, Michal Ramot, Guido Hesselmann, Audrey Vanhaudenhuyse, Ilan Dinstein, Jean-Flory L. Tshibanda, Melanie Boly, Michal Harel, Steven Laureys, Rafael Malach

**Affiliations:** 1 Department of Neurobiology, Weizmann Institute of Science, Rehovot, Israel; 2 Berlin School of Mind and Brain, Humboldt University, Berlin, Germany; 3 Department of Psychiatry, University of Wisconsin-Madison, Madison, Wisconsin, United States of America; 4 Coma Science Group, Cyclotron Research Center and Neurology department, University of Liège, Liège, Belgium; 5 Department of Psychiatry, Charité Campus Mitte, Berlin, Germany; Cuban Neuroscience Center, Cuba

## Abstract

Clinical diagnosis of disorders of consciousness (DOC) caused by brain injury poses great challenges since patients are often behaviorally unresponsive. A promising new approach towards objective DOC diagnosis may be offered by the analysis of ultra-slow (<0.1 Hz) spontaneous brain activity fluctuations measured with functional magnetic resonance imaging (fMRI) during the resting-state. Previous work has shown reduced functional connectivity within the “default network”, a subset of regions known to be deactivated during engaging tasks, which correlated with the degree of consciousness impairment. However, it remains unclear whether the breakdown of connectivity is restricted to the “default network”, and to what degree changes in functional connectivity can be observed at the single subject level. Here, we analyzed resting-state inter-hemispheric connectivity in three homotopic regions of interest, which could reliably be identified based on distinct anatomical landmarks, and were part of the “Extrinsic” (externally oriented, task positive) network (pre- and postcentral gyrus, and intraparietal sulcus). Resting-state fMRI data were acquired for a group of 11 healthy subjects and 8 DOC patients. At the group level, our results indicate decreased inter-hemispheric functional connectivity in subjects with impaired awareness as compared to subjects with intact awareness. Individual connectivity scores significantly correlated with the degree of consciousness. Furthermore, a single-case statistic indicated a significant deviation from the healthy sample in 5/8 patients. Importantly, of the three patients whose connectivity indices were comparable to the healthy sample, one was diagnosed as locked-in. Taken together, our results further highlight the clinical potential of resting-state connectivity analysis and might guide the way towards a connectivity measure complementing existing DOC diagnosis.

## Introduction

The coupling between conscious awareness and its external motor manifestation is so pervasive that it is difficult to comprehend the devastating state of fully conscious patients who are unable to respond. Severe brain injury can lead to such cases, termed the “locked-in” syndrome (LIS). As a result of motor disconnection, it is challenging to differentiate such cases from those in which awareness itself is disrupted – termed vegetative state (VS) or minimally conscious state (MCS). The differential diagnosis between VS and MCS is even more challenging and up to 40% misdiagnosis has been reported [1], [2], [3], [4]. The method of choice for diagnosis of conscious status has been careful bedside observations, which are challenging due to fluctuation in arousal, motor deficits and other deficits attributed to the injury, such as aphasia. This method, due to its subjective nature could partly contribute to the misdiagnosis rate [5]. Recent studies have demonstrated that fMRI may provide some DOC patients with a means for communication through blood oxygen level dependent (BOLD) signals evoked by mental imagery, even in the complete absence of motor outputs [6], [7]. However, this method relies on patient cooperation as well as attentional capacity and may not be suitable for the general patient population. Even more problematic is prognosis, the ability to predict which patients have better chances of recovery. These challenges highlight the urgent need for an objective physiological measure complementing current evaluation tools.

Recently, a series of studies uncovered a robust phenomenon that offers exciting potential for a complementary diagnosis of unresponsive patients. Even in the absence of intentional sensory-motor tasks, the human cortex manifests high-amplitude ultra-slow (<0.1 Hz) fluctuations in its BOLD signals that reflect distinct functional systems [8], [9], [10]. These spontaneous fluctuations show anatomical specificity in that correlations (also termed functional-connectivity) are more pronounced between the functionally related cortical regions (e.g. right and left auditory cortices) than between functionally unrelated cortical regions (e.g. ‘Extrinsic’/‘task-positive’ and ‘default-mode’ networks [11]). A particularly striking and consistent aspect of this connectivity is the correlation across homotopic sites in the two hemispheres [9], [12], [13], [14]. More recently, a likely neuronal correlate of these spontaneous BOLD fluctuations was found in ultra-slow modulations of neuronal firing rates and gamma power in local field potentials [12], [15], [16].

Although the functional role of the ultra-slow spontaneous fluctuations remains unclear, they could potentially aid clinical diagnosis. Indeed, such fluctuations and their network correlations were shown to be altered in several neurological and neuropsychiatric disorders [10], [17], [18], [19], [20]. The spontaneous nature of ultra-slow fluctuations, emerging without the need for intentional cooperation, makes them ideally suited as a complementary diagnostic tool in DOC. It has been recently shown that connectivity within the default-network [21], a subset of regions that are deactivated during externally oriented tasks, is negatively correlated with the degree of clinical consciousness impairment [22], see also [23]. In these studies resting–state connectivity within the default mode network (DMN) was assessed using probabilistic independent component analysis in DOC patients and an alteration in the spatial extent of the DMN was found at the group level [22] and at the individual level [23].

Although it has been suggested that the DMN is associated with basic functions related to consciousness [24], such as self-related processes [25], [26], it is not clear whether the reduction in connectivity is restricted to the default-mode network or rather extends into externally oriented regions. Furthermore, in order to use connectivity analyses for the diagnosis of these patients, it will have to be established how reliable these measures are on a subject-by-subject basis.

Here, we examined resting-state inter-hemispheric connectivity in three homotopic regions of interest, which were easily identified based on anatomical landmarks, and were part of the externally oriented network. We found reduction in inter-hemispheric functional connectivity in impaired awareness subjects as compared to intact awareness subjects. In addition, functional connectivity was correlated with the level of consciousness and was found to deviate from the healthy sample in in 5/8 patients using a single case statistical test. Importantly, of the three patients whose connectivity indices were comparable to the healthy sample, one was diagnosed as locked-in. These results suggest that resting-state functional connectivity might prove beneficial in the future as a complementary measure in the diagnosis of DOC patients.

## Materials and Methods

The study was approved by the ethics committee of the Faculty of Medicine at the University of Liège, Belgium. Written informed consent for healthy volunteers and patients was obtained from all subjects and legal guardians, respectively.

### Subjects

Nineteen subjects participated in the study. Eleven healthy subjects with no neurological or psychiatric history were recruited (age 28.8±4.5 years). Eight neurological patients (age 53.4±16.7 years) were evaluated using the CRS-R scale [27], and a diagnosis of locked-in syndrome (LIS, n = 1), minimally conscious state (MCS, n = 2), vegetative state (VS, n = 2), coma (n = 2), or brain death (BD, n = 1) was established. The LIS subject was diagnosed using the CRS-R and the FOUR scales [28]. BD diagnosis was established when CRS-R testing showed no brain stem reflexes, and was further confirmed by a physician conducting apnea tests [29] as well as EEG recordings [30]. Recovery was assessed using the Glasgow Outcome Scale, GOS [31]. The etiology of brain injuries was traumatic (n = 1), anoxic (n = 2), due to cerebral vascular accidents (CVA, n = 2), hemorrhagic (n = 1), meningitis (n = 1), or meningioma (n = 1). See Table S1 for further clinical details.

### Data

The data used for this project were also used for two other published studies that have applied different methods of analysis and addressed functional connectivity in different cortical networks. Data from fifteen subjects were used in a study published by Vanhaudenhuyse and colleagues [22], which addressed default network connectivity using Independent Component Analysis. The brain dead subject has also been analyzed in a study by Boly and colleagues [32], who addressed functional connectivity in the default mode network. Data from the three remaining subjects (two controls and one MCS patient) were not used in any previously published work.

### Functional imaging

Functional magnetic resonance imaging (fMRI) data were obtained in a 10 minute resting-state scan using a Siemens Tim Trio 1.5T scanner at the University Hospital Centre CHU-Sart Tilman in Liège, Belgium. Healthy subjects were instructed to lie still and keep their eyes closed for the duration of the scan, with no overt task being imposed. No sedation was applied in patients. Three-dimensional functional images using blood oxygen level dependent (BOLD) contrast were obtained with a gradient echo planar imaging (EPI) sequence (TR = 3000 ms, TE = 30 ms, 36 slices; voxel size: 3.75×3.75×3.6 mm, flip angle 90°). T1-weighted anatomical images were acquired using a 3D MPRAGE sequence (TR = 1670 ms, TE = 4.5 ms, TI = 1000 ms, 144 slices, voxel size: 1.2×0.9×1.4 mm, flip angle 8°). Subjects with excessive head motion (>1 mm translation, >1 deg rotation) were excluded from the analysis; nine subjects (7 patients; 2 healthy controls) considered for MRI were excluded during acquisition due to excessive movement in the scanner. DOC patients tend to exhibit involuntary movements due to increased muscle tone. As our patients were not sedated during the MR scan, we had to exclude 7 patients from the analysis.

### fMRI preprocessing

FMRI data were preprocessed using BrainVoyager QX 1.9 (Brain Innovation, Maastricht, The Netherlands) and complementary software written in MATLAB R2009b (The MathWorks, USA). The first two images of each functional scan were discarded to avoid T1 saturation effects. Preprocessing of functional scans included 3D motion correction, linear trend removal, and spatial smoothing using a Gaussian filter kernel of 8 mm full-width-at-half-maximum (FWHM). For all further analysis, data were band-pass filtered between 0.01 and 0.08 Hz. Twenty volumes were removed from the beginning and the end of the scan to avoid edge artifacts induced by the filtering, leaving 158 volumes for the analysis. Several sources of spurious variance were removed from the signal time-course of each voxel through linear regression [33]: 1) the average signal from each subject's ventricles, 2) the average signal from each subject's white matter voxels, and 3) the average signal from each subject's grey matter voxels (“global signal”). Data were normalized to the Talairach coordinate system [34], and the cortical surface was reconstructed for each subject as described previously [35]. Inflated and flattened cortical maps were used to visualize statistical parametric maps.

### ROI definitions

We focused on long-distance inter-hemispheric correlations because these are less susceptible to local noise sources, such as local blood flow modulations [8], [12]. Regions of interest (ROIs) serving as “seeds” for the inter-hemispheric correlations were manually identified in the pre- and post-central gyrus (preCG, postCG) and in the intra-parietal sulcus (IPS) of the right hemisphere. This choice of ROIs was guided by multiple considerations in addition to the goal of assessing connectivity outside the DMN. First, previous studies indicate that inter-hemispheric correlations are reliably observed in these regions [14], [36]. Second, correlations between regions on the lateral cortical mantle were less affected by correlated noise due to shared vascular supply, movement artifacts, or spatial spread of midline signals [12]. Third, and most importantly, we anticipated the potential use of such analysis in routine clinical procedures and thus selected regions that are easily identifiable through simple anatomical landmarks.

PostCG and preCG ROIs were defined based on the localization of the “hand knob” on the central sulcus, known to be largely consistent with the motor hand area [37]. Areas from the gyri anterior and posterior to this knob were defined as preCG (209.4±45.9 voxels, isotropic voxel size: 3×3×3 mm) and postCG (208.4±43 voxels) ROIs, respectively. The ROI in IPS (83.9±31.9 voxels) was defined based on the intersection of the post-central sulcus with the intra-parietal sulcus [38]. In addition, we confirmed that the results were robust to the precise delineation of the ROIs, by demonstrating a tight correlation with results based on ROIs marked by an independent researcher (see Material S1 for further details).

### fMRI data analysis

For each subject, three resting-state functional connectivity maps were computed within the framework of the General Linear Model [39], using “seed” time-courses sampled from right hemisphere ROIs as regressors (preCG, postCG, IPS). Fits to the model were evaluated after removing the auto-regression factor [40]. Unless otherwise stated, the resulting statistical parametric maps were thresholded at p<0.01. Correction for multiple comparisons at the cluster level was performed using the AlphaSim plugin for BrainVoyager QX [41]. Subjects were divided into two groups based on their level of awareness [42]: the ‘intact awareness’ group comprising healthy and LIS subjects and the ‘impaired awareness’ group comprising MCS, VS, coma and brain-dead patients. Second-level statistical analysis across subjects within each group was performed using a random-effects analysis. The resulting three resting-state functional connectivity group maps were projected on an inflated and flattened 3D reconstruction of the cortical surface. The difference between the group maps was evaluated using two-sample t-tests. Thus, the resulting t-test maps (Figure S2) reflect regions that show significantly different resting-state functional connectivity in the two groups. Note that the two-sample t-test accounts for the different sample sizes of the two groups by weighting the variance terms. The t-test maps were thresholded at p<0.05. Finally, an inter-hemispheric correlation index (ICD) was computed for each subject by averaging across the three inter-hemispheric correlations of spontaneous BOLD fMRI activity between homotopic ROIs (i.e., left and right postCG/preCG/IPS). To test for a relationship between individual ICD values and the level of consciousness, we calculated the non-parametric Spearman correlation coefficient. To test whether individual ICD scores from patients significantly deviate from the healthy subjects (used as normative sample), we applied a t-test specifically developed for single case studies [43]. This modified t-statistic by Crawford & Howell tests for the rarity or abnormality of a patient's score, using the standard deviation of a group of healthy subjects (as the normative sample of size N) as an estimate for the population standard deviation and N-1 degrees of freedom.

## Results

**Figure 1:**
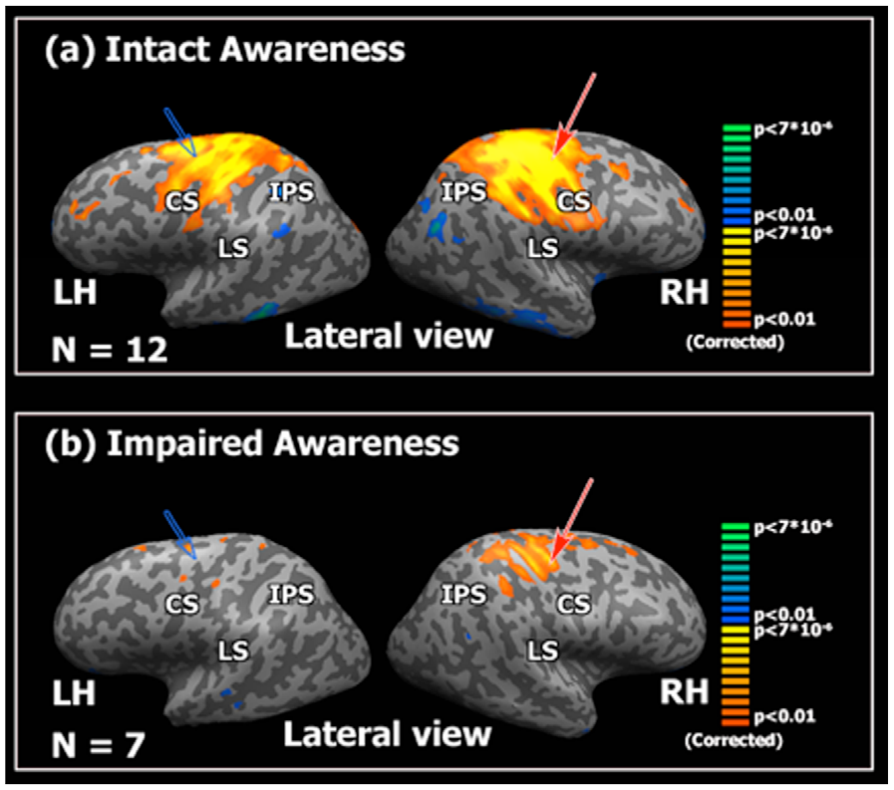
Correlations between spontaneous BOLD fluctuations in right pre-central gyrus and other cortical regions. Group correlation maps between a “seed” region in the pre-central gyrus (preCG) and all other cortical voxels, projected on inflated left (LH) and right (RH) hemispheres (lateral view). (a) Correlations of spontaneous activity in the intact awareness group (n = 12). (b) Correlations in the impaired awareness group (n = 7). Red arrow, “seed” region location. Blue arrow, homotopic “mirror” regions in the left hemisphere. Note that inter-hemispheric correlations are largely absent in the impaired awareness group. Abbreviations: CS, central sulcus; LS, lateral sulcus; IPS, intra-parietal sulcus.

We examined correlations between spontaneous BOLD activity in selected cortical regions of interest (ROIs) of the “Extrinsic”- externally oriented, task-positive network [11], [44] and all other cortical regions. Subjects were tentatively grouped into intact and impaired awareness (see methods for further details). Figure 1 shows the correlation map computed for a “seed” ROI in the right preCG. In the intact awareness group (Figure 1A), activity in the preCG significantly correlated with neighboring somato-sensory cortex, and with the homotopic “mirror” region in the left hemisphere. Activity in the cingulate sulcus was also significantly correlated with that of the preCG (Figure S1). Negative correlations were observed in the posterior cingulate cortex, lateral temporal cortex, and inferior parietal lobule, which are commonly referred to as the default-mode network [45], [46] or “intrinsic” network [11], [25]. In the impaired awareness group, significant correlations were restricted to immediately neighboring cortex, while inter-hemispheric correlations were largely absent (Figure 1B). This drastic reduction of inter-hemispheric correlations was confirmed by statistically comparing the group maps (Figure S2) taking into account the different sample sizes. Highly similar results were observed for “seed” regions in the right intra-parietal sulcus (IPS, Figure S3) and the right posterior central gyrus (postCG, Figure S4).

**Figure 2:**
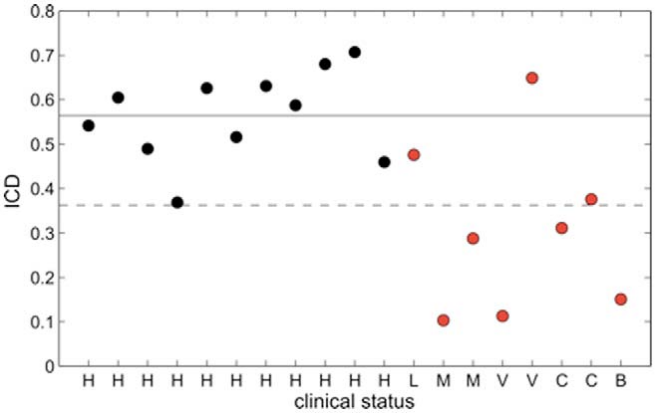
Inter-hemispheric Correlation Index (ICD) in individual subjects. Subjects are separated on the x-axis depending on their clinical state (patients in red and healthy controls in black). The solid line represents the mean ICD value in the healthy controls group and the dashed line represents the mean-2*standard deviation. Abbreviations: H, healthy; L, locked-in syndrome; V, vegetative state; M, minimally conscious state; C, coma; B, brain death.

To obtain a quantitative measure of the inter-hemispheric correlations in each subject, we introduced an inter-hemispheric correlation index (ICD). This index represents the average inter-hemispheric correlations between the pre-defined ROIs (for details see materials and methods). We found that ICD values were decreased in the majority of DOC patients (Figure 2). Individual ICD scores were found to significantly correlate with the degree of consciousness, ranging from brain dead, coma, VS, MCS, LIS, to healthy controls (Spearman's correlation coefficient r = .61, p = .0057). Since a decrease of resting-state connectivity with age has been reported for the DMN [[47]; but see [48]], we confirmed that lower ICD values in the patient group did not reflect age differences. Indeed, the correlation between age and ICD value for all participants was not statistically significant. (Spearman's correlation coefficient r = −.36, p = .128). In addition, when using age as a control variable in a partial correlation analysis, the correlation between ICD and the level of consciousness remained significant (r = .52, p = .027). We further addressed this concern by analyzing data from additional 11 healthy controls that were matched for age (53.54±15.97 years). Resting-state data and structural scans were downloaded from a freely available online source (http://fcon_1000.projects.nitrc.org/indi/pro/nki.html), and identical preprocessing and analysis was performed (see Figure S5, Table S2 and Material S2 for further details). The results show that when individual ICD values of DOC patients were tested against the normative sample of age-matched controls (see below), highly similar statistical results were obtained as found in the younger control group.

**Figure 3:**
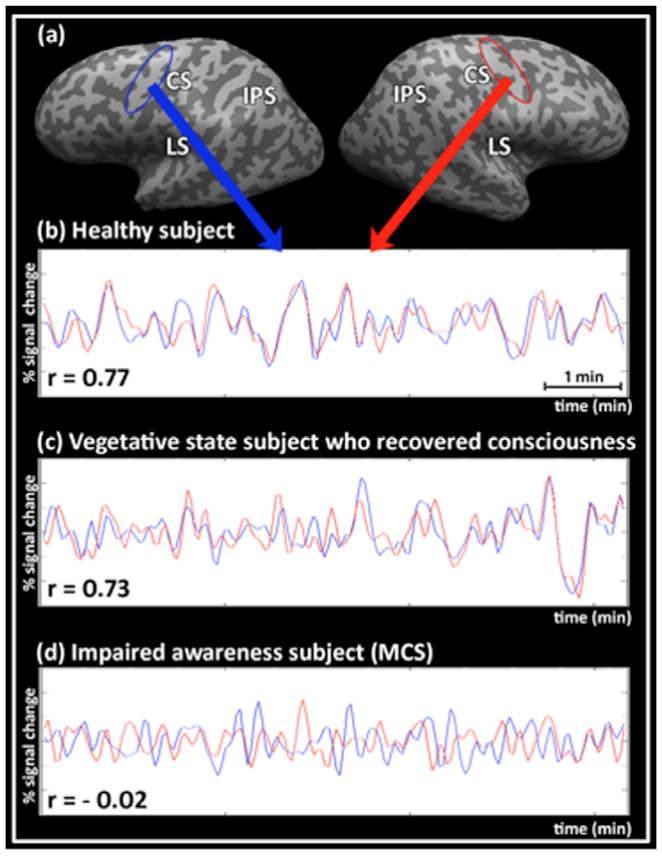
Correlations between spontaneous BOLD signal time-courses across hemispheres. (a) Anatomical locations of regions-of-interest (ROIs) in the right pre-central gyrus (red arrow) and the homotopic ROI in the left hemisphere (blue arrow). (b) Time-courses in a healthy subject exhibit high correlation (r = 0.77). (c) Time-courses in the vegetative patient who recovered consciousness exhibit high correlation (r = 0.73). (d) Time-courses in an impaired awareness patient exhibit low correlation (r = −0.02). Red and blue time-courses denote signals from the right and left hemispheres, respectively normalized to percent signal change. Abbreviations: CS, central sulcus; LS, lateral sulcus; IPS, intra-parietal sulcus.

To test whether individual ICD scores from patients significantly deviate from the healthy subjects (used as normative sample), we used a t-test specifically developed for single case studies [43]. As shown in Table 1, individual ICD values from 5/8 patients were significantly different (p<.05) from the average ICD value of the healthy controls. In addition to the ICD measure, the disruption of inter-hemispheric correlations in DOC patients could also be discerned to a large extent in individual BOLD time courses (Figure 3) and single-subject maps (Figure S6).

**Table 1:** ICD values and Crawford and Howell test results.

Consciousness level	ICD value	t(10)	p – value
LIS	0.4757	−0.8417	0.2098
MCS1	0.1033	−4.3682	0.0007*
MCS2	0.2877	−2.6219	0.0128*
VS1	0.1126	−4.2799	0.0008*
VS2	0.6489	0.798	0.2217
Coma1	0.3113	−2.3987	0.0187*
Coma2	0.3753	−1.7923	0.0517
Brain Death	0.1506	−3.9196	0.0014*
Control1	0.3683	-	-
Control2	0.4592	-	-
Control3	0.4894	-	-
Control4	0.5156	-	-
Control5	0.5415	-	-
Control6	0.5875	-	-
Control7	0.6047	-	-
Control8	0.6261	-	-
Control9	0.6308	-	-
Control10	0.6803	-	-
Control11	0.7072	-	-

Taken together, these results demonstrate that impaired awareness was associated with reduced inter-hemispheric correlations and was largely evident at the single subject level. The three patients with non-significant difference from the healthy control group were the LIS patient (which was expected to have normal ICD since his consciousness level is identical to a healthy control), one Coma patient (which showed a trend towards significance, p = .0517) and one VS patient (which would be expected to have decreased correlation values). The ICD value of this VS patient, studied seven days after anoxic brain damage was 0.65, well within the range of healthy controls (0.56±0.1; mean ± SD). Importantly, shortly after the brain imaging (13 days), this patient progressed to a state of MCS and later (40 days post scan), recovered consciousness, reaching a state of “moderate disability” according to the Glasgow Outcome Scale [31]. In our sample, this was the only patient to improve in diagnosis, (see Glasgow Outcome Scale for all patients in Table S1), one patient remained in a MCS and the rest of the patients did not survive.

Importantly, inter-operator variability was low; control analysis based on ROIs drawn by a separate fMRI researcher yielded a significant correlation (r = 0.65, p<0.01, Figure S7) between the ICD scores obtained by these two independent investigators, supporting of the future potential of this method for clinical use (see Table S3 and Material S1 for further information).

## Discussion

In this study, we show reduced inter-hemispheric connectivity between homologous cortical regions within the “Extrinsic”, task positive network using resting-state fMRI in DOC patients. The observed decrease in connectivity was significantly correlated with the degree of consciousness impairment, and was evident to a large extent at the single subject level.

Inter-hemispheric correlations, or symmetry, between homologous regions is one of the prominent characteristics of resting-state fMRI and have been demonstrated in healthy populations using various techniques, such as region of interest based analysis [9], [44], independent component analysis (ICA) [14], [36], and whole brain approaches [49], [50]. Inter-hemispheric symmetry appears to be a ubiquitous characteristic of brain anatomy [51] and function [52]. Inter-hemispheric connections, in analogous fashion to within-hemisphere connections, play a role in the integration of information and coordination between the two hemispheres. Not surprisingly, alteration in inter-hemispheric correlation has been demonstrated in various diseases and behavioral impairments [53], [54], [55], [56], [57] illustrating the importance of intactness of communication/synchronization for the normal functioning of the brain. Although the link between reduction in connectivity within the DMN and DOC has been shown previously [22], [23], it has remained unclear whether a reduction in connectivity is restricted to this network alone. Our results demonstrate a significant reduction in connectivity between homotopic regions belonging to the “Extrinsic”, task positive network. This finding raises the question of how widespread the reduction of connectivity is in DOC patients. Since reduction in connectivity is not restricted to one network, it seems, however, that DOC might reflect a more global impairment in functional connectivity and in the integrity of different circuits. In other words, it may be that reduced connectivity within specific cortical networks may affect specific behaviors but is not sufficient to affect the overall level of consciousness. Along this line, it has been shown in stroke patients that a reduction in connectivity that is specific to the attention network is reflected behaviorally as neglect symptoms, but not in the level of consciousness [58]. Indeed, reduction in connectivity within one network might not be a sufficient marker for the diagnosis of DOC patients, and a whole brain analysis might be better suited to test more global impairments.

The exact source of the reduction in connectivity is still not fully understood. However, it is beyond the scope of our paper to investigate whether the striking disruption in the inter-hemispheric correlations observed in DOC patients is due to a widespread cortical, subcortical or white matter damage. Although structural damage will evidently lead to loss of connectivity, especially in the immediate time following injury [59], but see [60] and [61], such reduction may be linked to synaptic changes well below the resolution of brain imaging and could also be influenced by plasticity changes following the injury through sub-cortical connections [60]. In addition, reduction in functional connectivity following structural damage has been reported for areas that appear structurally intact [58], [62]. Recently, Bruno et al. reported a case of “functional hemispherectomy” in two DOC patients, with near-normal DMN components in one hemisphere, given a structural and metabolic deficit in the other hemisphere [63]. This result highlights the need for a multimodal neuroimaging approach, as one of the challenges related to connectivity measures in the population of DOC patients will be to conceive a quantification of the heterogeneous damage typically observed in this population, taking into account asymmetric structural damage which poses a challenge for the ICD measure proposed in our study.

On a more general scope, the inference one can make about the cognitive level from spontaneous fMRI fluctuations remains controversial. On the one hand, spontaneous fluctuations can appear in the absence of any task or intentional activity, are detectable during anesthesia [64], [65], and even accentuated during sleep [12], [66]. On the other hand, other evidence implicates a contribution of ultra-slow fluctuations in perceptual decision making [67], [68] and motor control [69]. Furthermore, the fact that the spatial organization of spontaneous ultra-slow activity replicates task-related activity of functional networks [8], [9], [14] suggests that their presence may reflect a hebbian co-activation process [70], and conversely, their disruption may thus be due to a reduction in network functionality.

As to the clinical significance of our findings, the results point to the potential usefulness of the ICD in diagnosing individual cases of impaired awareness. In order to validate such measure for future clinical use, a larger population of DOC patients and healthy subjects need to be tested, thus also allowing for an estimation of the ICD measure's specificity and sensitivity. The fact that we did not observe the lowest ICD value in the brain dead patient further emphasizes the need for a larger sample to separate “true” ICD values from ICD values generated by spurious noise. In a recent paper by Boly and colleagues, using a seed based approach in the default mode network, a brain dead patient failed to show any significant correlations in a whole brain map [32].

The unexpected recovery of the VS patient showing a normal-level ICD, points to the promising possibility that the ICD index may serve, at least in specific cases, as a prognostic measure for recovery from DOC. However, this single observation is of course far from providing a conclusive demonstration and should be taken at this stage only as a catalyst for a wide-scope search for additional similar cases.

To conclude, we propose the ICD index as a measure of symmetry in functional connectivity that can be used as a diagnostic marker in DOC. This measure has the advantage of relying on spontaneous fMRI signal fluctuations and thus does not depend on patient cooperation, which is often absent in DOC patients.

## Supporting Information

Material S1 **Inter-operator variability analysis.**
(DOC)

Material S2 **Comparison of the ICD to an aged-matched control group.**
(DOCX)

Figure S1 **Correlations between spontaneous BOLD fluctuations in right pre-central gyrus and all other cortical regions.** Group correlation maps between a “seed” region in the pre-central gyrus (preCG) and all other cortical voxels. (a) Correlations of spontaneous activity in the intact awareness group (n = 12) projected on inflated hemispheres as seen from a lateral view (top left) and a medial view (top right), as well as a flat format (bottom). (b) Correlations in the impaired awareness group (n = 7). Format as above. Red arrow, “seed” region location. Blue arrow, “mirror” regions in the left hemisphere. Note that inter-hemispheric correlations are largely absent in the impaired awareness group. Abbreviations: LH, left hemisphere; RH, right hemisphere; CS, central sulcus; LS, lateral sulcus; IPS, intra-parietal sulcus; CinS, cingulate sulcus; POS, parieto-occipital sulcus.
(PNG)

Figure S2 **Voxel-by-voxel differences in Spontaneous BOLD correlations between intact- and impaired-awareness groups.** Statistical maps of two-sample t-tests (see Methods) comparing BOLD signal correlations in the two subject groups (intact, n = 12; impaired, n = 7) separately for each voxel. Maps are projected on inflated cortical surfaces as seen from lateral (top) and medial (bottom) views in each panel. Panels show differences in BOLD correlations of spontaneous activity with a “seed” in the (a) right intra-parietal sulcus, (b) right post-central gyrus, and (c) right pre-central gyrus. Note that in all maps, significant differences were found in contralateral “mirror” sites (yellow ellipses in the left hemisphere), as well as in the vicinity of seed regions. Abbreviations: LH, left hemisphere; RH, right hemisphere; IPS, intra-parietal sulcus; CS, central sulcus.
(PNG)

Figure S3 **Correlations between spontaneous BOLD fluctuations in right intra-parietal sulcus and all other cortical regions.** Group correlation maps between a “seed” region in the intra-parietal sulcus (IPS) and all other cortical voxels. (A) Correlations of spontaneous activity in the intact awareness group (n = 12) projected on inflated hemispheres as seen from a lateral view (top left) and a medial view (top right), as well as a flat format (bottom). (B) Correlations in the impaired awareness group (n = 7). Format as above. Red arrow, “seed” region location. Blue arrow, “mirror” regions in the left hemisphere. Note that inter-hemispheric correlations are largely absent in the impaired awareness group. Abbreviations: LH, left hemisphere; RH, right hemisphere; CS, central sulcus; LS, lateral sulcus; IPS, intra-parietal sulcus; CinS, cingulate sulcus; POS, parieto-occipital sulcus.
(PNG)

Figure S4 **Correlations between Spontaneous BOLD fluctuations in right post-central gyrus and all other cortical regions.** Group correlation maps between a “seed” region in the post-central gyrus (postCG) and all other cortical voxels. (A) Correlations of spontaneous activity in the intact awareness group (n = 12) projected on inflated hemispheres as seen from a lateral view (top left) and a medial view (top right), as well as a flat format (bottom). (B) Correlations in the impaired awareness group (n = 7). Format as above. Red arrow, “seed” region location. Blue arrow, “mirror” regions in the left hemisphere. Note that inter-hemispheric correlations are largely absent in the impaired awareness group. Abbreviations: LH, left hemisphere; RH, right hemisphere; CS, central sulcus; LS, lateral sulcus; IPS, intra-parietal sulcus; CinS, cingulate sulcus; POS, parieto-occipital sulcus.
(PNG)

Figure S5 **Inter-hemispheric Correlation Index (ICD) in individual subjects in all three groups.** Subjects are separated on the x-axis depending on their group (controls1: non-aged matched group; controls2: aged-matched group, and patients). Abbreviations: (*) refers to the VS patient who regained consciousness shortly after scan (VS2 in the supplementary tables), (+) refers to the Locked-in patient.
(TIF)

Figure S6 **Single subject inter-hemispheric correlation maps (seed: right PreCG) ordered according to the ICD values.** Correlation maps with a “seed” time-course in the right pre-central gyrus (pre-CG) are shown in flat, left hemisphere (“mirror site”) cortical format for each subject separately. (a) Location of seed (red ellipse) and location of the “mirror site” in the contra-lateral hemisphere (black arrow). (b) Intact awareness group, (c) Impaired awareness group. Abbreviations: LH, left hemisphere; RH, right hemisphere; CS, central sulcus; LS, lateral sulcus; I, individual ICD value; VS*, vegetative state patient who recovered consciousness shortly following our study; LIS, locked-in syndrome.
(PNG)

Figure S7 **Inter-operator correlation of ICD measure.** ICD values as computed on a subsample of 11 subjects by two independent operators drawing the ROIs. Abbreviations: CCC, concordance correlation coefficient; r, Pearson correlation.
(TIF)

Table S1 **Clinical, electrophysiological and structural imaging data of patients.** Abbreviations: LIS, locked-in syndrome; VS, vegetative state; MCS, minimally conscious state.
(DOCX)

Table S2 **ICD values and Crawford and Howell test results for the age-matched sample.** Abbreviations: LIS, locked-in syndrome; VS, vegetative state; MCS, minimally conscious state.
(DOCX)

Table S3 **Inter-operator variability data.** Abbreviations: IPS, intra-parietal sulcus; preCG, pre-central gyrus; postCG, post-central gyrus.
(DOCX)
