# Supplementary material for: Reduction in Inter-Hemispheric Connectivity in Disorders of Consciousness
Source: PLoS One. 2012 May 22;7(5):e37238. doi: 10.1371/journal.pone.0037238 (PMC3358327; doi:10.1371/journal.pone.0037238)
Supplement: Material S1 — Inter-operator variability analysis. (DOC) [file pone.0037238.s001.doc]

*Inter-operator variability:*

In order to address the issue of inter-operator variability, we have reanalyzed a subset of our data (n=11) using ROIs marked by an independent researcher. Instructions regarding the location of the anatomical landmarks were provided, including the hand knob area of the central sulcus and the intra-parietal sulcus in its intersection with the post central sulcus. Regions were marked on the transverse plane using a 3D drawing tool available in BrainVoyager (Brain Innovation, Maastricht, The Netherlands), as done by the first operator. The gyrus anterior to the central sulcus was marked (the PreCG), the gyrus posterior to the central sulcus was marked (PostCG), and the intra-parietal sulcus (IPS) was marked, both in the left and in the right hemisphere. Additional information on the average ROI size was provided in order to prevent large differences in ROIs sizes. However, the size of ROIs for individual subjects was not provided in order to allow the operator to take specific individual anatomy into account. The final result of ICD values was compared between the two operators.

As can be seen in Figure S7, the two operators had a good agreement with a significant Pearson correlation coefficient of 0.65 (p < 0.05). Significant correlations were found for all three ROIs comprising the ICD measure (IPS: r = 0.73 p = 0.01; PostCG: r = 0.92, p < 0.001; PreCG: r = 0.67 p = 0.02). In addition, a concordance correlation coefficient was computed, as described by Lin and colleagues , which yielded a concordance value of 0.643. Table S3 provides the information regarding size and center coordinates of the regions drawn by the two operators. No significant difference in size of the ROIs was found between the two operators. In conclusion, the low inter-operator variability further supports the potential clinical use of the ICD method described in our paper.

71. Lin LI (1989) A concordance correlation coefficient to evaluate reproducibility. Biometrics 45: 255-268.
