# Supplementary material for: Reduction in Inter-Hemispheric Connectivity in Disorders of Consciousness
Source: PLoS One. 2012 May 22;7(5):e37238. doi: 10.1371/journal.pone.0037238 (PMC3358327; doi:10.1371/journal.pone.0037238)
Supplement: Material S2 — Comparison of the ICD to an aged-matched control group. (DOCX) [file pone.0037238.s002.docx]

*Comparison of the ICD to an aged-matched control group:*

In order to confirm that our main finding of lower ICD values in the patients group did not reflect age differences, we have obtained data from 11 additional healthy controls matched for age in the patient group (age 53.54 ± 15.97 years). Resting-state data and structural scans were downloaded from a freely available online source for resting-state fMRI, (<http://fcon_1000.projects.nitrc.org/indi/pro/nki.html>). fMRI data were obtained in a 10.8 minute (260 volumes) resting-state scan using a Siemens Tim Trio 3T scanner at the Nathan Kline Institute for Psychiatric Research. Three-dimensional functional images using blood oxygen level dependent (BOLD) contrast were obtained with a gradient echo planar imaging (EPI) sequence (TR = 2500 ms, TE = 30 ms, 38 slices; voxel size: 3x3x3 mm, 0.33 mm gap, flip angle 80°). T1-weighted anatomical images were acquired using a 3D MPRAGE sequence (TR = 2500 ms, TE = 3.5 ms, TI = 1200 ms, 190 slices, voxel size: 1x1x1 mm, flip angle 8°). No excessive head motions (>1 mm translation, >1 deg rotation) were recorded in any of the subjects. Preprocessing was identical to the preprocessing performed on the original dataset (see materials and methods). In addition, matching of the time points (volumes of scan) between the samples was performed, leaving 158 volumes for the analysis. ICD was computed for each individual subject as described in the materials and methods. A computation of the Crawford & Howell t-test was performed using the aged–matched group as the normative sample (see Table S2).

The results show that when individual ICD values of DOC patients were tested against the normative sample of age-matched controls, highly similar statistical results were obtained as previously reported for a younger control group (see Figure S5). Importantly, the VS patient who regained consciousness shortly after the scan had an ICD value that was not significantly different from healthy controls (see Table S2). The ICD value for the locked-in patient turned out to be significant at the .01 significance level. However, the exact p-value (p = .0053) was one magnitude larger than that of the maximal p-value observed for the remaining DOC patients (p = .0007). These results strongly suggest that the decreased ICD values observed in the DOC patients were not due to age difference between the groups.
