# Supplementary figures and images for: Reduction in Inter-Hemispheric Connectivity in Disorders of Consciousness
Source: PLoS One. 2012 May 22;7(5):e37238. doi: 10.1371/journal.pone.0037238 (PMC3358327; doi:10.1371/journal.pone.0037238)

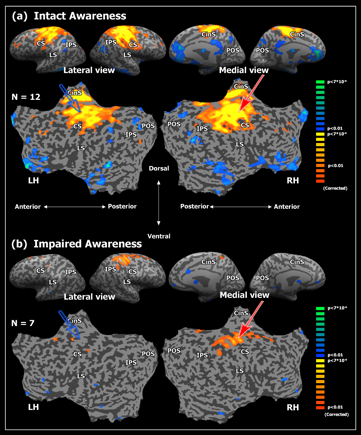

Supplement: Figure S1 — Correlations between spontaneous BOLD fluctuations in right pre-central gyrus and all other cortical regions. Group correlation maps between a “seed” region in the pre-central gyrus (preCG) and all other cortical voxels. (a) Correlations of spontaneous activity in the intact awareness group (n = 12) projected on inflated hemispheres as seen from a lateral view (top left) and a medial view (top right), as well as a flat format (bottom). (b) Correlations in the impaired awareness group (n = 7). Format as above. Red arrow, “seed” region location. Blue arrow, “mirror” regions in the left hemisphere. Note that inter-hemispheric correlations are largely absent in the impaired awareness group. Abbreviations: LH, left hemisphere; RH, right hemisphere; CS, central sulcus; LS, lateral sulcus; IPS, intra-parietal sulcus; CinS, cingulate sulcus; POS, parieto-occipital sulcus. (PNG) [file pone.0037238.s003.png]

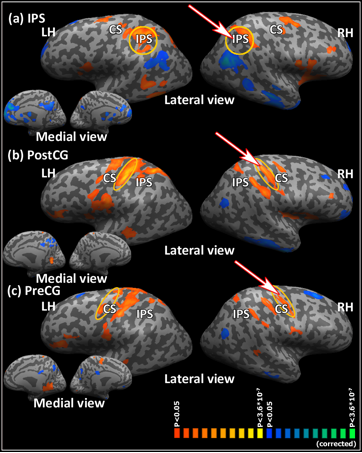

Supplement: Figure S2 — Voxel-by-voxel differences in Spontaneous BOLD correlations between intact- and impaired-awareness groups. Statistical maps of two-sample t-tests (see Methods) comparing BOLD signal correlations in the two subject groups (intact, n = 12; impaired, n = 7) separately for each voxel. Maps are projected on inflated cortical surfaces as seen from lateral (top) and medial (bottom) views in each panel. Panels show differences in BOLD correlations of spontaneous activity with a “seed” in the (a) right intra-parietal sulcus, (b) right post-central gyrus, and (c) right pre-central gyrus. Note that in all maps, significant differences were found in contralateral “mirror” sites (yellow ellipses in the left hemisphere), as well as in the vicinity of seed regions. Abbreviations: LH, left hemisphere; RH, right hemisphere; IPS, intra-parietal sulcus; CS, central sulcus. (PNG) [file pone.0037238.s004.png]

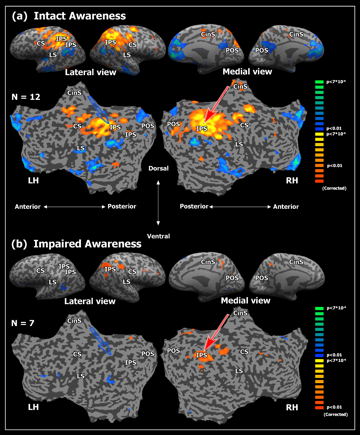

Supplement: Figure S3 — Correlations between spontaneous BOLD fluctuations in right intra-parietal sulcus and all other cortical regions. Group correlation maps between a “seed” region in the intra-parietal sulcus (IPS) and all other cortical voxels. (A) Correlations of spontaneous activity in the intact awareness group (n = 12) projected on inflated hemispheres as seen from a lateral view (top left) and a medial view (top right), as well as a flat format (bottom). (B) Correlations in the impaired awareness group (n = 7). Format as above. Red arrow, “seed” region location. Blue arrow, “mirror” regions in the left hemisphere. Note that inter-hemispheric correlations are largely absent in the impaired awareness group. Abbreviations: LH, left hemisphere; RH, right hemisphere; CS, central sulcus; LS, lateral sulcus; IPS, intra-parietal sulcus; CinS, cingulate sulcus; POS, parieto-occipital sulcus. (PNG) [file pone.0037238.s005.png]

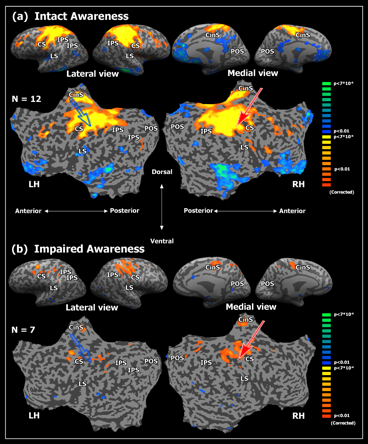

Supplement: Figure S4 — Correlations between Spontaneous BOLD fluctuations in right post-central gyrus and all other cortical regions. Group correlation maps between a “seed” region in the post-central gyrus (postCG) and all other cortical voxels. (A) Correlations of spontaneous activity in the intact awareness group (n = 12) projected on inflated hemispheres as seen from a lateral view (top left) and a medial view (top right), as well as a flat format (bottom). (B) Correlations in the impaired awareness group (n = 7). Format as above. Red arrow, “seed” region location. Blue arrow, “mirror” regions in the left hemisphere. Note that inter-hemispheric correlations are largely absent in the impaired awareness group. Abbreviations: LH, left hemisphere; RH, right hemisphere; CS, central sulcus; LS, lateral sulcus; IPS, intra-parietal sulcus; CinS, cingulate sulcus; POS, parieto-occipital sulcus. (PNG) [file pone.0037238.s006.png]

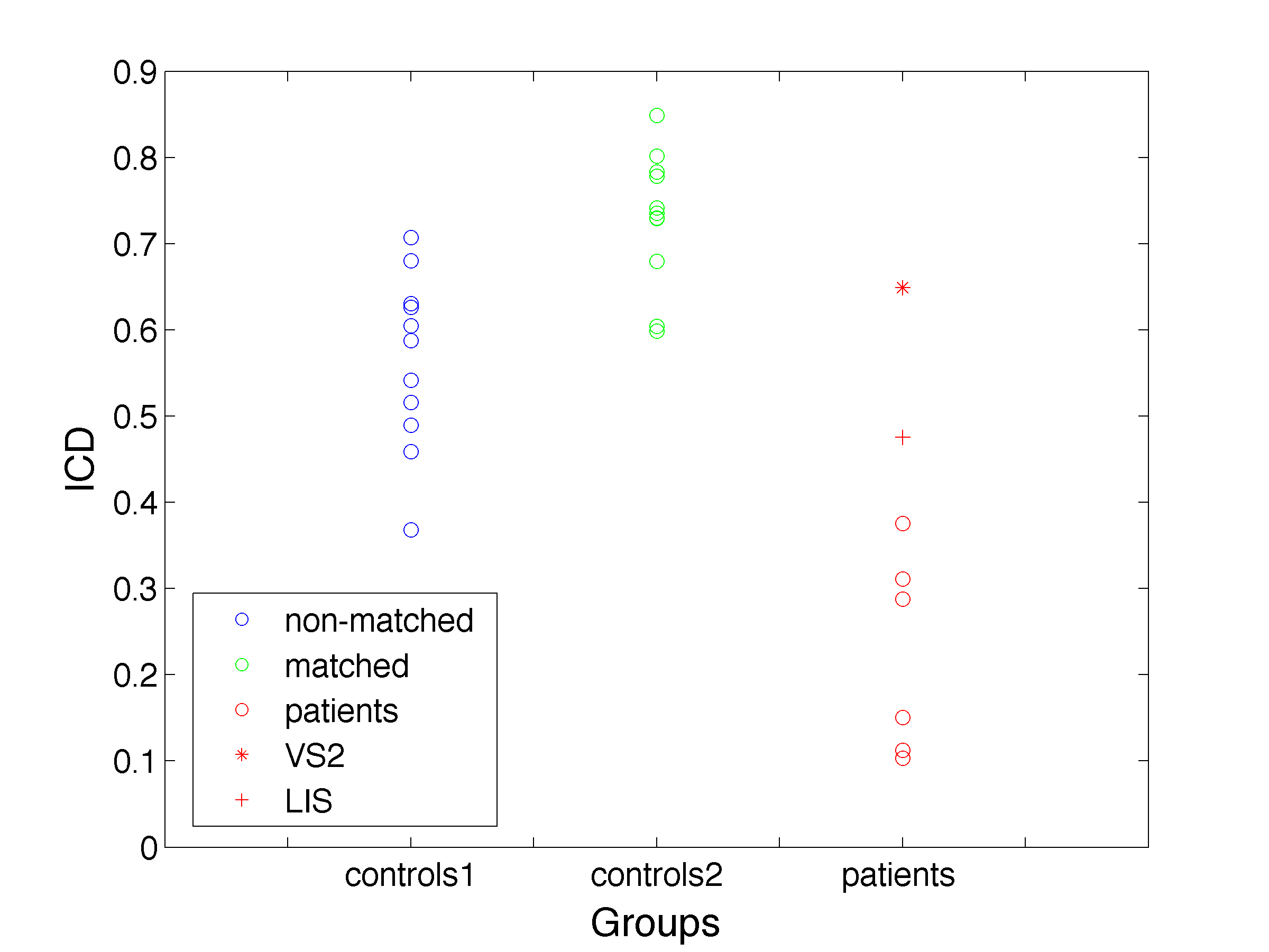

Supplement: Figure S5 — Inter-hemispheric Correlation Index (ICD) in individual subjects in all three groups. Subjects are separated on the x-axis depending on their group (controls1: non-aged matched group; controls2: aged-matched group, and patients). Abbreviations: (*) refers to the VS patient who regained consciousness shortly after scan (VS2 in the supplementary tables), (+) refers to the Locked-in patient. (TIF) [file pone.0037238.s007.tif]

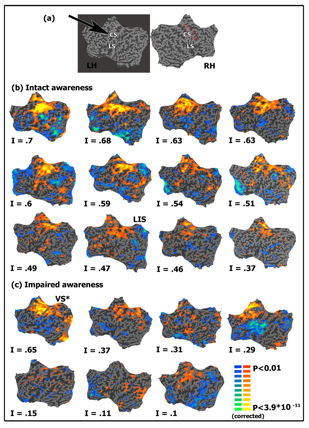

Supplement: Figure S6 — Single subject inter-hemispheric correlation maps (seed: right PreCG) ordered according to the ICD values. Correlation maps with a “seed” time-course in the right pre-central gyrus (pre-CG) are shown in flat, left hemisphere (“mirror site”) cortical format for each subject separately. (a) Location of seed (red ellipse) and location of the “mirror site” in the contra-lateral hemisphere (black arrow). (b) Intact awareness group, (c) Impaired awareness group. Abbreviations: LH, left hemisphere; RH, right hemisphere; CS, central sulcus; LS, lateral sulcus; I, individual ICD value; VS*, vegetative state patient who recovered consciousness shortly following our study; LIS, locked-in syndrome. (PNG) [file pone.0037238.s008.png]

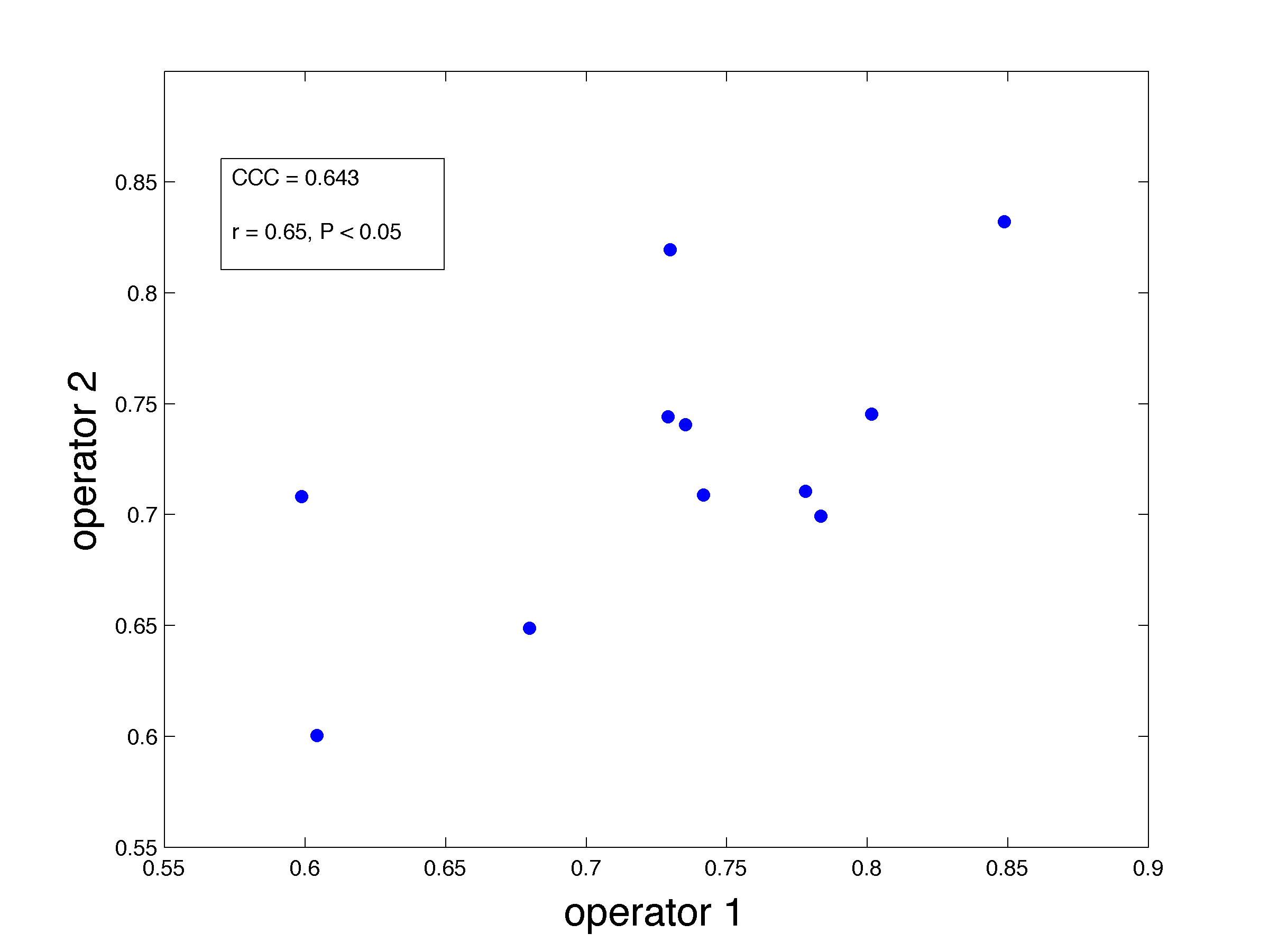

Supplement: Figure S7 — Inter-operator correlation of ICD measure. ICD values as computed on a subsample of 11 subjects by two independent operators drawing the ROIs. Abbreviations: CCC, concordance correlation coefficient; r, Pearson correlation. (TIF) [file pone.0037238.s009.tif]
