# Supplementary material for: Reduction in Inter-Hemispheric Connectivity in Disorders of Consciousness
Source: PLoS One. 2012 May 22;7(5):e37238. doi: 10.1371/journal.pone.0037238 (PMC3358327; doi:10.1371/journal.pone.0037238)
Supplement: Table S1 — Clinical, electrophysiological and structural imaging data of patients. Abbreviations: LIS, locked-in syndrome; VS, vegetative state; MCS, minimally conscious state. (DOCX) [file pone.0037238.s010.docx]

**10. Table S1. Clinical, electrophysiological and structural imaging data of patients.**

|  | **LIS** | **MCS1** | **MCS2** | **VS1** | **VS2** | **COMA1** | **COMA2** | **Brain Dead** |
| --- | --- | --- | --- | --- | --- | --- | --- | --- |
| **Clinical Features** |  |  |  |  |  |  |  |  |
| Sex (age, years) | Female (48) | Male (29) | Female (65) | Male (70) | Male (36) | Female (78) | Female (51) | Female (50) |
| Cause | Cerebro-Vascular Accident | Trauma | Meningitis | Anoxia | Anoxia | Cerebro-Vascular Accident | Meningioma | Haemorrhage |
| Time of fMRI  (days after insult) | 16 | 4.3 years | 14 | 36 | 7 | 13 | 14 | 8 |
| Outcome at 12 months (Glasgow Outcome Scale) | Dead (1) | Severe disability (3) | Dead (1) | Dead (1) | Moderate disability (4) | Dead (1) | Dead (1) | Dead (1) |
| Breathing | Spontaneous | Spontaneous | Spontaneous | Spontaneous, with tube | Spontaneous | Assisted breathing | Spontaneous | Controlled breathing |
| **Coma Recovery Scale-Revised** |  |  |  |  |  |  |  |  |
| Diagnosis at time of fMRI | Locked-In Syndrome | Minimally Conscious State | Minimally Conscious State | Vegetative State | Vegetative State | COMA | COMA | Brain Dead |
| Auditory function | Systematic movement to command | Reproducible movement to command | Reproducible movement to command | None | None | Startle reflex | None | None |
| Visual function | Object recognition | Visual pursuit | Visual pursuit | None | Blink reflexes | None | None | None |
| Motor function | Flexion to pain | Flexion to pain | None | None | Flexion to pain | Flexion to pain | Flexion to pain | None |
| Oromotor/Verbal function | Oral reflexes | Oral reflexes | Vocalisations | Oral reflexes | None | Oral reflexes | None | None |
| Communication | Functional | None | None | None | None | None | None | None |
| Arousal | Without stimulation | Without stimulation | Without stimulation | With stimulation | Without stimulation | None | None | None |
| Total score | 17 | 11 | 10 | 2 | 5 | 4 | 2 | 0 |
| **EEG** |  |  |  |  |  |  |  |  |
| Background activity | Bilateral slow delta | NA | Delta-theta irregular | Diffuse slow delta activity | Symetric slow theta activity | Diffuse slow theta and delta activity | Diffuse slow theta activity | Isoelectric |
| **MRI** |  |  |  |  |  |  |  |  |
| Increased intensity on T2 | Bulbo-medullar and cerebral peduncle lesions | Cerebral peduncle atrophy. Right frontal, temporal and motor area and left semi-oval centre diffuse axonal lesions | Supratentorial lesions | Sylvian ischemic and brainstem lesions | Lateral ventricular and right internal capsule lesions | Left thalamus and left mesencephalic lesions | Protuberantial and bulbo-medullar lesions | Diffuse brain swelling consecutive to the vasospasm, with  diffuse white matter damage and hemorrhagic sequela  predominant in the medial frontal lobe |
