# Supplementary material for: Reduction in Inter-Hemispheric Connectivity in Disorders of Consciousness
Source: PLoS One. 2012 May 22;7(5):e37238. doi: 10.1371/journal.pone.0037238 (PMC3358327; doi:10.1371/journal.pone.0037238)
Supplement: Table S2 — ICD values and Crawford and Howell test results for the age-matched sample. Abbreviations: LIS, locked-in syndrome; VS, vegetative state; MCS, minimally conscious state. (DOCX) [file pone.0037238.s011.docx]

**Table S2:**

ICD values and Crawford and Howell test results for the age-matched sample.

| **Consciousness level** | **ICD value** | **t(10)** | **p - value** |
| --- | --- | --- | --- |
| LIS | 0.4757 | -3.1385 | 0.0053* |
| MCS1 | 0.1033 | -7.7355 | <0.0001* |
| MCS2 | 0.2877 | -5.4592 | 0.0001* |
| VS1 | 0.1126 | -7.6205 | <0.0001* |
| VS2 | 0.6489 | -1.0011 | 0.1702 |
| Coma1 | 0.3113 | -5.1683 | 0.0002* |
| Coma2 | 0.3753 | -4.3778 | 0.0007* |
| Brain Death | 0.1506 | -7.1508 | <0.0001* |
| Control12 | 0.7291 | - | - |
| Control13 | 0.8014 | - | - |
| Control14 | 0.8487 | - | - |
| Control15 | 0.7298 | - | - |
| Control16 | 0.7835 | - | - |
| Control17 | 0.7353 | - | - |
| Control18 | 0.7780 | - | - |
| Control19 | 0.7416 | - | - |
| Control20 | 0.6041 | - | - |
| Control21 | 0.6797 | - | - |
| Control22 | 0.5986 | - | - |
