# Supplementary material for: Reduction in Inter-Hemispheric Connectivity in Disorders of Consciousness
Source: PLoS One. 2012 May 22;7(5):e37238. doi: 10.1371/journal.pone.0037238 (PMC3358327; doi:10.1371/journal.pone.0037238)
Supplement: Table S3 — Inter-operator variability data. Abbreviations: IPS, intra-parietal sulcus; preCG, pre-central gyrus; postCG, post-central gyrus. (DOCX) [file pone.0037238.s012.docx]

**Table S3:** Inter-operator variability data.

|  | **Operator 1** | | **Operator 2** | |
| --- | --- | --- | --- | --- |
|  | Size  (Mean ± std) | Center Coordinates  (Mean) | Size  (Mean ± std) | Center Coordinates  (Mean) |
| Right IPS | 80.9 ± 14.8 | (31, -52, 39) | 77.9 ± 8 | (31, -59, 40) |
| Right PostCG | 210.6 ± 16.9 | (35, -31, 52) | 221.4 ± 5.4 | (38, -30, 50) |
| Right PreCG | 205.1 ± 22.9 | (32, -21, 51) | 212 ± 12.7 | (34, -19, 51) |
| Left IPS | 81.9 ± 14.8 | (-32, -51, 39) | 81.1 ± 9.3 | (-33, -58, 40) |
| Left PostCG | 211.3 ± 15.5 | (-37, -31, 52) | 210.5 ± 11.2 | (-40, -30, 50) |
| Left PreCG | 206.4 ± 22.9 | (-35, -20, 50) | 211.2 ± 11.2 | (-36, -18, 50) |
